# Psychological and Behavioural Correlates of Dietary Intake in Adolescent Elite Female Handball Players

**DOI:** 10.3390/nu18172904

**Published:** 2026-09-04

**Authors:** Zsófia Sziráki, Diána Führer, Lajos Biró, Zsófia Kosik, Márta Szmodis

**Affiliations:** 1 Department of Health Sciences and Sport Medicine, Hungarian University of Sports Science, 1123 Budapest, Hungary; diafuhrer@gmail.com (D.F.); szmodis.marta@tf.hu (M.S.); 2 Department of Dietetics and Nutritional Science, Faculty of Health Sciences, Semmelweis University, 1088 Budapest, Hungary; biro.lajos@semmelweis.hu; 3 Bethesda Children’s Hospital, 1146 Budapest, Hungary; zsofia.kosik@gmail.com

**Keywords:** adolescent athletes, eating behaviour, dietary intake, sport anxiety, resilience, female athletes, handball, psychological factors, nutrition

## Abstract

**Background/Objectives:** Adequate dietary intake is essential for supporting growth, health, and athletic performance in adolescent athletes. Handball is a high-intensity intermittent team sport characterised by repeated running, sprinting, jumping, and physical contact, placing substantial physiological and nutritional demands on young athletes. This study examined associations between eating behaviour dimensions, sport anxiety, resilience, and dietary intake in adolescent elite female handball players. **Methods:** This cross-sectional study included 74 adolescent elite female handball players; after exclusion of two dietary-intake outliers, 72 participants were included in the analyses (mean age: 15.6 ± 1.7 years; BMI: 21.2 ± 2.4 kg/m^2^). Eating behaviour, sport anxiety, and resilience were assessed using the TFEQ-R21, SAS-2, and CD-RISC-10, respectively. Dietary intake was estimated using a semi-quantitative food frequency questionnaire. Pearson correlation and linear regression analyses were performed. **Results:** Cognitive Restraint was negatively correlated with energy (*r* = −0.266, *p* = 0.024), fat (*r* = −0.288, *p* = 0.014), carbohydrate (*r* = −0.257, *p* = 0.029), and sugar intake (*r* = −0.255, *p* = 0.031). Uncontrolled Eating was positively associated with energy (β = 0.338, *p* = 0.030; *R*^2^ = 0.134) and fat intake (β = 0.366, *p* = 0.017; *R*^2^ = 0.157) in the regression analyses. Higher resilience was associated with lower sugar intake in the regression analysis (β = −0.318, *p* = 0.007; model R^2^ = 0.101) and with lower sport anxiety in the Pearson correlation analysis (*r* = −0.559, *p* < 0.001). **Conclusions:** The findings indicate associations between eating behaviour dimensions, selected psychological characteristics, and dietary intake in adolescent elite female handball players. Uncontrolled Eating and Cognitive Restraint showed distinct patterns of association with dietary intake, while resilience was associated with sugar intake and sport anxiety. Given the cross-sectional and exploratory nature of the study, these findings warrant cautious interpretation and replication in larger independent samples.

## 1. Introduction

Adolescence represents a critical developmental period during which health-related behaviours are established and may have long-term consequences for future health [1]. Evidence suggests that obesity-related behaviours, including dietary habits, show moderate tracking from childhood and adolescence to adulthood, highlighting the importance of understanding factors that influence eating behaviours during this life stage [2]. Consequently, identifying behavioural determinants of dietary intake during adolescence may have implications not only for current health and performance but also for long-term health trajectories. Adequate energy and nutrient intake are of fundamental importance for adolescent athletes, not only to support growth and development but also to meet the increased energy demands associated with regular training and competition. Therefore, nutritional intake must simultaneously support healthy development and optimise athletic performance [3,4,5]. These considerations may be particularly relevant in handball, a high-intensity intermittent team sport that places substantial demands on both aerobic and anaerobic energy systems and involves repeated high-intensity actions such as sprinting, jumping, throwing, and changes in direction. Accordingly, adequate energy and carbohydrate availability may be particularly important for supporting the demands of training and competition in adolescent handball players. Notably, female adolescent handball players have been reported to display more restrictive dietary habits than their male counterparts [6]. These sex-related differences further highlight the relevance of investigating dietary behaviours specifically in adolescent female handball players.

The relevance of focusing specifically on adolescent female athletes is further supported by evidence that eating-related vulnerabilities and their correlates may differ between adolescent and adult female athletes [7].

Eating behaviours are shaped by a complex interaction of biological, psychological, social, and environmental factors and play an important role in food choices and dietary patterns throughout childhood and adolescence [8]. Recent evidence further supports the role of eating behaviour traits in shaping dietary habits. A systematic review of studies in children and adolescents reported consistent associations between eating behaviour characteristics and food group consumption, dietary choices, and overall dietary patterns [9]. In addition, prospective evidence from the Generation XXI birth cohort suggests that appetitive traits assessed during childhood are associated with subsequent food consumption patterns, indicating that eating behaviour characteristics may influence later dietary choices [10]. Consequently, examining eating behaviour characteristics may provide a more comprehensive understanding of dietary intake patterns in adolescent athletes.

Food choice, eating-related motivations, and dietary practices can be examined through eating behaviour. According to the Three-Factor Eating Questionnaire (TFEQ), eating behaviour can be described along three major dimensions: Uncontrolled Eating, Emotional Eating, and Cognitive Restraint. Individuals characterized by uncontrolled eating exhibit a tendency to overeat due to a perceived loss of control over eating and heightened responsiveness to external food-related cues, such as the sight, smell, or availability of food. Emotional eating refers to an increased tendency to consume food in response to emotional states rather than physiological hunger. Cognitive restraint represents the conscious restriction of food intake in order to control body weight or body shape [11]. However, a reduction in self-control, particularly during periods of negative emotional states, may impair eating regulation and contribute to excessive food intake [12].

The relationship between eating behaviour dimensions and energy intake has been relatively underexplored in the scientific literature. One international study by Anschutz et al. [13] investigated the associations between eating behaviour dimensions and dietary intake among female university students using the Dutch Eating Behavior Questionnaire (DEBQ). Although the present study employed the Three-Factor Eating Questionnaire (TFEQ-R21), both instruments assess conceptually related eating behaviour dimensions. Anschutz et al. reported that external eating was positively associated with total energy, fat, and carbohydrate intake, whereas restrained eating showed inverse associations with these dietary variables after controlling for BMI and physical activity. No significant association was found between emotional eating and energy intake.

In Hungarian studies, associations have been reported between eating behaviour dimensions and several psychological and behavioural correlates [14,15]. In female college students, associations between eating behaviour and food preference were weak but showed the expected pattern: Cognitive Restraint was associated with lower consumption of energy-dense foods, whereas Uncontrolled Eating and Emotional Eating were associated with higher consumption of energy-dense foods [14].

The relationship between body size and dietary intake appears to be complex. In a large population-based study of German adults, associations between BMI and macronutrient intake differed across the BMI distribution, suggesting that dietary intake patterns cannot be fully explained by body size alone [16]. Furthermore, recent evidence from the HELENA study demonstrated that eating behaviour characteristics, including cognitive restraint and emotional eating, are associated with body composition in European adolescents, highlighting the relevance of behavioural factors alongside anthropometric indicators when examining nutrition-related outcomes [17]. Taken together, these findings suggest that higher BMI or greater adiposity may be associated with greater conscious efforts to regulate food intake across different age groups. Consequently, eating behaviour characteristics may provide information relevant to dietary intake beyond anthropometric indicators alone.

Eating behaviour is influenced by several psychological factors, among which stress and anxiety play a particularly important role. Stress can disrupt the body’s internal balance (homeostasis), thereby threatening both physical and psychological well-being. Anxiety is considered one of the primary responses to stress [18] and is closely related to multiple dimensions of mental health, including emotional, psychological, and social well-being [19]. Anxiety can be expressed in both somatic (e.g., physiological symptoms) and cognitive (e.g., worry, concentration difficulties) components, which allow it to be operationalized and examined in research [18]. In athletic populations, sport participation can have several positive psychological effects, such as improved self-awareness, increased self-confidence, enhanced concentration, and greater life satisfaction. However, high physical demands, performance pressure, competitive situations, and social interactions (e.g., coach–athlete, athlete–athlete relationships) may also contribute to increased stress levels, which can in turn lead to anxiety [18].

Resilience is a multidimensional characteristic that refers to an individual’s ability to adapt successfully to adversity, recover from setbacks, and maintain psychological well-being despite challenging circumstances, and may serve as an important psychological resource against stress- and anxiety-related difficulties [20]. It has been identified as an important psychological resource that may protect young athletes from the negative effects of stress and anxiety.

Research has consistently demonstrated a negative association between resilience and anxiety among adolescent athletes. González-Hernández et al. [21] found that youth athletes with higher levels of resilience reported lower levels of competitive anxiety. The authors suggested that resilient athletes possess greater emotional regulation skills, self-confidence, and coping resources, which help them interpret stressful situations as manageable challenges rather than overwhelming threats. Similarly, Wang [22] reported that resilience significantly predicted lower levels of pre-competition anxiety among adolescent table tennis players, indicating that resilience may serve as a protective factor against performance-related stress. However, both studies used different measures of resilience and different forms of anxiety than those employed in the present study. To our knowledge, no studies have directly examined the association between resilience measured using the Connor–Davidson Resilience Scale (CD-RISC) and sport anxiety measured using the Sport Anxiety Scale-2 (SAS-2) in adolescent athletes. Therefore, investigating the associations between these two constructs may contribute novel evidence regarding the role of resilience in sport-specific anxiety.

Regarding athletes, research has primarily focused on the relationship between anxiety and eating disorders, with evidence consistently demonstrating a positive association between the two [23,24]. Hungarian research examining the psychological determinants of eating behaviour has identified self-control and motivation as significant predictors of food-choice patterns among secondary school students [25].

Despite growing evidence regarding eating behaviour, BMI, anxiety, and dietary intake, relatively few studies have simultaneously examined eating behaviour traits, sport anxiety, resilience, and dietary intake in adolescent female athletes. Furthermore, evidence regarding psychological predictors of energy and macronutrient intake in team sport athletes remains limited.

Therefore, the aim of the present study was to examine the associations between eating behaviour dimensions, sport anxiety, resilience, BMI, and dietary intake in adolescent elite female handball players. Specifically, the study examined the associations of eating behaviour dimensions, sport anxiety, and resilience with daily energy, macronutrient, and sugar intake. In addition, given the lack of studies simultaneously examining resilience and sport-specific anxiety in adolescent athletes using the CD-RISC and SAS-2, the study also explored the associations between resilience and sport anxiety.

## 2. Materials and Methods

### 2.1. The Participants

This study focused on a relatively homogeneous sample of adolescent female athletes. Recruitment was facilitated by the team coaches, who initially informed the players about the study. Participants were recruited using convenience sampling as part of a broader research project involving adolescent elite female handball players. Thus, the selection of handball players was primarily determined by the availability of this population within the broader research framework rather than by sport-specific sampling. All players within the eligible age groups of the participating teams were invited to participate; no individual selection of players was made by the coaches or researchers. The written study information and consent/assent documentation explicitly stated that participation was voluntary and that participants could decline or withdraw without consequences. The total number of players who received the initial invitation and the reasons for non-participation were not systematically recorded; therefore, the number of eligible non-participants and those who actively declined participation cannot be reliably determined retrospectively. The study included 74 adolescent elite female handball players from the youth teams of two Hungarian first-division clubs (Siófok KC, Moyra-Budaörs Handball Club). Participants were aged between 12.5 and 20.4 years, with a mean age of 15.6 ± 1.7 years. In the final analytical sample (*N* = 72), the large majority of the sample were minors: sixty-six participants (91.7%) were younger than 18 years, while six participants (8.3%) were aged 18 years or older. All participants were secondary-school students, including those aged 18 years or older, who were attending Grades 12 or 13. Thus, despite the relatively broad chronological age range, participants shared a broadly similar educational and sporting environment. The World Health Organization defines adolescence as the period between 10 and 19 years of age [26]. However, broader developmental definitions extending adolescence to 24 years have also been proposed [27]. In the present sample, only one participant was older than 20 years (20.4 years), while all other participants were younger than 20 years. Mean body weight was 60.2 ± 8.8 kg, mean height was 168.0 ± 6.5 cm, and mean BMI was 21.2 ± 2.4 kg/m^2^.

### 2.2. Procedure

Data collection was conducted on two designated assessment days between June and October 2023. As the participants were recruited from two handball clubs located in different Hungarian cities, data collection was conducted separately at each club. Participants from one club (*n* = 38) were assessed in June 2023. They arrived in the morning following an overnight fast. Body composition measurements were performed first, with participants wearing light clothing (underwear only). Following the body composition assessment, participants were allowed to eat and drink as usual. The SQFFQ was administered first, followed by brief sociodemographic questions, the Hungarian Satisfaction with Life Scale (SWLS-H), the CD-RISC-10, the Hungarian Mental Health Continuum–Short Form (MHC-SF-H), the SAS-2, the TFEQ-R21, and brief questions on physical activity. The SWLS-H and MHC-SF-H were administered as part of the broader psychological assessment battery but were not included in the analyses of the present study, as they were not directly related to the specific research aims.

Participants from the other club (*n* = 36) were assessed in October 2023 during the early afternoon. Due to the timing of data collection, body composition measurements were not performed in this subgroup. Participants completed a light training session and were subsequently given the opportunity to eat before completing the questionnaires. They completed the questionnaires in the same predefined order and under the same standardised completion conditions as participants at the first assessment.

No competitive match was scheduled for either group on the day of assessment.

To facilitate the accurate identification and classification of food items, particularly for younger participants, study-specific visual aids were provided during completion of the SQFFQ. These materials included photographs of commonly available food products and typical Hungarian brands and packaging, helping participants distinguish between food categories that they might not have been able to identify based on product characteristics alone. The visual aids were primarily intended to support food identification and classification; standardised photographic portion-size information was not systematically provided for all food items. A registered dietitian (MSc) was available throughout the assessment to answer questions and provide clarification when needed. Participation was voluntary, and participants were free to withdraw from the study at any time without consequences. A team coach was present at the beginning of each assessment session, introduced the researchers to the participants, and remained present during the study briefing. The coach was also present at the end of the assessment session. Questionnaires were completed individually, with one empty seat maintained between participants to ensure independent responding. Participants were given sufficient time to complete the questionnaires without time pressure. Two researchers were present throughout questionnaire administration to provide clarification when requested. Before submission, questionnaires were checked for completeness, and participants were asked to complete any inadvertently omitted items. Questionnaires were not scored or clinically evaluated during the assessment sessions; the on-site review was limited to checking response completeness.

### 2.3. Methods

#### 2.3.1. Dietary Intake

Dietary intake was assessed using a semi-quantitative food frequency questionnaire (SQFFQ) developed specifically for the Hungarian population by Lajos Biró, one of the authors of the present study. Participants were instructed to report the frequency and usual portion size of foods and beverages that generally characterised their dietary intake. The questionnaire comprised 90 food and beverage items grouped into 17 major food categories. The questionnaire was developed using data from the Hungarian food composition database. To facilitate the accurate identification and classification of food items, particularly for younger participants, study-specific visual aids were provided during completion of the SQFFQ. These materials included photographs of commonly available food products and typical Hungarian brands and packaging, helping participants distinguish between food categories that they might not have been able to identify based on product characteristics alone. The visual aids were used to support food identification; in a limited number of cases, they also provided supplementary support for portion-size estimation. The completed questionnaires were analysed using the NutriComp-SQFFQ 2.0 software. The software estimates daily energy and nutrient intake using calculation algorithms developed for NutriComp dietary assessment programs and data derived from the Hungarian food composition database. Daily energy intake (kcal) and macronutrient intake, including protein (g), fat (g), carbohydrate (g), and sugar (g), were calculated and used in the analyses.

The methodology underlying the NutriComp^®^-SQFFQ system has been described previously by Biró [28]; however, the specific 90-item SQFFQ used in the present study has not been formally validated against an independent reference dietary assessment method.

#### 2.3.2. Eating Behaviour

Eating behaviour was assessed using the Hungarian version of the Three-Factor Eating Questionnaire-Revised 21-item (TFEQ-R21). Participants were instructed to select the response that best reflected their eating habits and feelings of hunger. The questionnaire assesses three dimensions of eating behaviour: Cognitive Restraint, Uncontrolled Eating, and Emotional Eating. Higher scores on each TFEQ-R21 subscale indicate a greater tendency towards the corresponding eating behaviour. No established cut-off values are currently available for the Hungarian version of the TFEQ-R21, and normative data from comparable Hungarian adolescent elite athlete populations are lacking. The original instrument was developed by Tholin et al. [11] and the Hungarian version was validated by Czeglédi and Urbán [29]. Previous studies have also demonstrated the applicability of the TFEQ-R21 in adolescent populations [30,31,32]. The internal consistency of the TFEQ-R21 subscales was evaluated in the present sample and was found to be good, with Cronbach’s α and McDonald’s ω values of 0.80 and 0.81 for Uncontrolled Eating, 0.82 and 0.83 for Cognitive Restraint, and 0.82 and 0.82 for Emotional Eating, respectively.

#### 2.3.3. Sport Anxiety

Sport anxiety was assessed using a Hungarian translation of the Sport Anxiety Scale-2 (SAS-2). Participants were instructed to indicate the response that generally reflected their level of anxiety before or during competitions. The SAS-2 is a 15-item questionnaire measuring sport-specific trait anxiety across three dimensions: Somatic Anxiety, Worry, and Concentration Disruption. Items are rated on a 4-point Likert scale, with higher scores indicating greater sport anxiety. Each subscale score ranges from 5 to 20, with higher scores indicating greater sport-related anxiety in the respective dimension. No established cut-off values or normative data for Hungarian adolescent populations are currently available for the SAS-2. The original scale was developed and validated in child, adolescent, and adult athletes by Smith et al. [33]. The internal consistency of the instrument was evaluated in the present sample and was found to be good to excellent, with Cronbach’s α and McDonald’s ω values of 0.80 and 0.81 for Somatic Anxiety, 0.91 and 0.92 for Worry, 0.74 and 0.75 for Concentration Disruption, and 0.90 and 0.90 for the total scale, respectively. The total SAS-2 score and the three subscale scores were used in the analyses.

#### 2.3.4. Resilience

Psychological resilience was assessed using the Hungarian 10-item version of the Connor–Davidson Resilience Scale (CD-RISC-10) [34], which was developed to measure an individual’s ability to cope effectively with adversity, stress, and challenging life circumstances [20]. Participants were instructed to indicate the extent to which each statement applied to them during the past month. Items are rated on a 5-point Likert scale ranging from 0 (“not true at all”) to 4 (“true nearly all the time”), with higher scores indicating greater resilience. No universally established clinical cut-off values are available for the CD-RISC-10, and normative data from directly comparable Hungarian adolescent female athlete populations are lacking. However, Hungarian reference data are available from other youth populations. Módné Takács and Pogátsnik (2025) reported a mean CD-RISC-10 score of 26.05 ± 6.96 in a large mixed-sex sample (*N* = 3275) [35]. In a more sport-specific population, Kiss et al. (2026) reported a mean item score of 3.19 ± 0.52 among male junior ice hockey players from state-accredited Hungarian academies [36]. In the present sample, the CD-RISC-10 demonstrated satisfactory internal consistency (Cronbach’s *α* = 0.77; McDonald’s *ω* = 0.78).

#### 2.3.5. Anthropometric Characteristics

Body composition was assessed using multi-frequency bioelectrical impedance analysis (InBody 770; InBody Co., Ltd., Seoul, South Korea) performed at six frequencies and four measurement points. Measurements were conducted in the early morning following a 5-min rest period, with participants wearing light clothing (underwear only). Participants were instructed to refrain from eating and drinking prior to the assessment. Due to logistical constraints related to data collection, body composition measurements were available for only a subsample of the participants (*n* = 38).

#### 2.3.6. Statistical Analysis

Statistical analyses were performed using Jamovi software (version 2.7.33) [37], while sensitivity power analyses were conducted using G*Power (version 3.1.9.7) [38]. Descriptive statistics are presented as means and standard deviations (*SD*). Prior to statistical analyses, dietary intake data were screened for extreme observations. Potential outliers in estimated daily energy intake were assessed using the interquartile range (*IQR*) method, with values exceeding Q3 + 1.5 × *IQR* considered upper outliers. The distribution of variables was assessed using the Shapiro–Wilk test, as well as visual inspection of histograms and Q-Q plots. Several variables showed statistically significant deviations from normality. Pearson correlation analyses were used as the primary analyses to examine associations between dietary intake variables, eating behaviour dimensions, sport anxiety measures, and resilience, while Spearman rank correlations were additionally performed as sensitivity analyses to assess the robustness of the observed associations. The magnitude of correlations was interpreted according to Cohen’s (1988) guidelines, with absolute correlation coefficients of approximately 0.10, 0.30, and 0.50 considered small, medium, and large, respectively [39]. Multiple linear regression analyses were conducted to determine whether eating behaviour dimensions predicted energy, macronutrient and sugar intake, both in absolute terms and, for macronutrients, as percentages of total energy intake. Additional sensitivity analyses were performed by including BMI as a covariate in these models. To assess whether the observed associations were influenced by the relatively broad age range of the sample, additional sensitivity analyses were performed by repeating the regression models with chronological age included as a covariate. These analyses were conducted for total energy intake and for protein, fat, carbohydrate, and sugar intake expressed both as absolute amounts and, where applicable, as percentages of total energy intake. Additional regression models were performed to examine the association of sport anxiety (SAS-2 total score and subscales) and resilience with energy and macronutrient intake. Given the observed association between resilience and absolute sugar intake, an additional regression analysis was conducted with sugar intake expressed as a percentage of total energy intake to examine whether this association persisted after accounting for total energy intake. Regression assumptions were evaluated using residual plots, Q-Q plots, and variance inflation factor (VIF) values. A sensitivity power analysis was performed to evaluate the magnitude of effects that could be detected with the final analytical sample (*N* = 72). With α = 0.05 and 80% power, the minimum detectable two-tailed bivariate correlation was |*r*| = 0.323. For linear regression models, the minimum detectable effect size was *f*^2^ = 0.112 for one-predictor models, *f*^2^ = 0.160 for three-predictor models, and *f*^2^ = 0.178 for four-predictor models. These values correspond to approximately *R*^2^ = 0.101, 0.138, and 0.151, respectively. The internal consistency of the TFEQ-R21 subscales, CD-RISC-10, and SAS-2 subscales and total score was evaluated using Cronbach’s *α* and McDonald’s *ω* coefficients. Statistical significance was set at *p* < 0.05.

## 3. Results

### 3.1. Participant Characteristics

A total of 74 elite female handball players competing in the Hungarian National First Division youth championship were included in the study. Screening of estimated daily energy intake identified two upper outliers according to the *IQR* criterion (9732.78 and 11,905.35 kcal/day), both exceeding the upper threshold of 8008.80 kcal/day. These two observations were excluded from the dietary analyses, resulting in a final analytical sample of 72 participants. The highest energy intake retained in the analyses was 7472.34 kcal/day. Participant characteristics, including weekly handball training volume and participation in other organised sports and school-based physical education, are presented in Table 1. Body composition data were available for *n* = 38 participants.

### 3.2. Dietary Intake and Psychological Characteristics

Descriptive statistics for absolute dietary intake variables, eating behaviour dimensions, and psychological characteristics are presented in Table 2. When expressed relative to body weight, mean daily energy intake was 58.3 ± 28.5 kcal/kg/day, while protein, fat, and carbohydrate intakes were 1.95 ± 0.93, 2.59 ± 1.30, and 6.39 ± 3.16 g/kg/day, respectively. When expressed as a percentage of total energy intake, protein, fat, carbohydrate, and sugar contributed 13.9 ± 1.63%, 41.1 ± 3.98%, 45.1 ± 4.57%, and 10.2 ± 3.95% of the total energy intake, respectively.

### 3.3. Correlation Analyses

The associations between absolute dietary intake variables, eating behaviour dimensions, sport anxiety, and resilience were examined using Pearson correlation analyses, and the results are presented in Table 3.

Cognitive Restraint was negatively correlated with energy intake, fat intake, carbohydrate intake, and sugar intake. Uncontrolled Eating was positively correlated with fat intake.

A significant medium positive correlation was observed between BMI and Cognitive Restraint (*r* = 0.360, *p* = 0.002), indicating that athletes with a higher BMI tended to report greater dietary restraint. No significant associations were found between BMI and Uncontrolled Eating (*r* = −0.023, *p* = 0.849) or Emotional Eating (*r* = 0.143, *p* = 0.232).

Among the sport anxiety variables, only the SAS-2 total score and Concentration Disruption were significantly associated with dietary intake. The SAS-2 total score showed a small positive correlation with carbohydrate intake (*r* = 0.233, *p* = 0.049) and sugar intake (*r* = 0.265, *p* = 0.024). Similarly, Concentration Disruption showed a small positive correlation with sugar intake. No significant correlations were observed between Somatic Anxiety or Worry and the examined dietary intake variables. Furthermore, none of the sport anxiety dimensions were significantly associated with total energy, protein, or fat intake (all *p* > 0.05).

A significant medium negative correlation was observed between resilience and sugar intake in the Pearson analysis (*r* = −0.318, *p* = 0.007). No significant correlations were observed between resilience and total energy intake, protein intake, fat intake, uncontrolled eating, cognitive restraint, or emotional eating.

**Figure 1:**
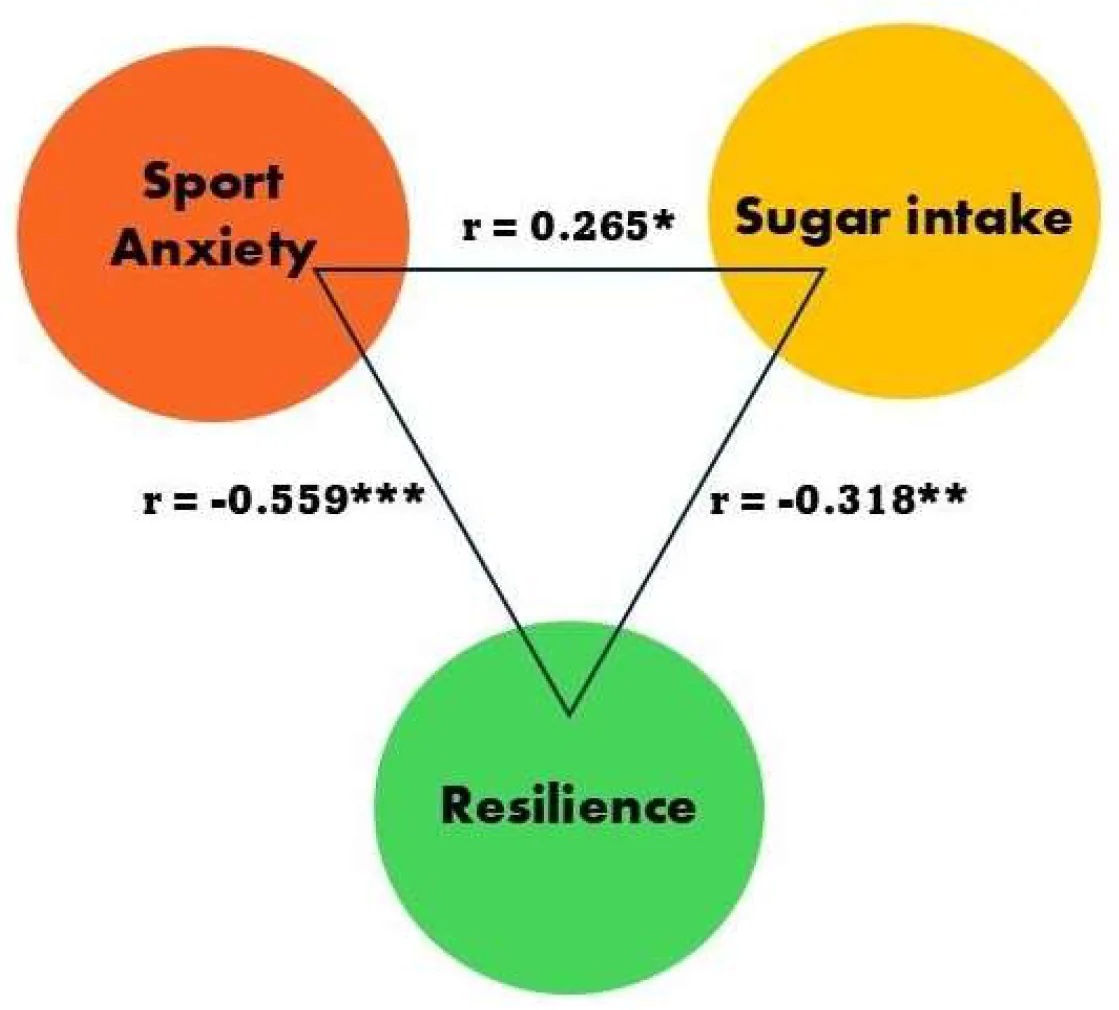
Significant associations identified in the Pearson correlation analyses among resilience, sport anxiety, and sugar intake in adolescent elite female handball players. Pearson correlation coefficients are displayed along the connecting lines. * *p* < 0.05, ** *p* < 0.01, *** *p* < 0.001.

Resilience was significantly and negatively correlated with sport anxiety and all of its dimensions. Higher resilience scores were associated with lower levels of overall sport anxiety (*r* = −0.559, *p* < 0.001), Concentration Disruption, Somatic Anxiety, and Worry. The pattern of significant associations among resilience, sport anxiety, and sugar intake is illustrated in Figure 1. No significant associations were observed between CD-RISC scores and the TFEQ-R21 dimensions, including Uncontrolled Eating, Cognitive Restraint, and Emotional Eating (all *p* > 0.05).

Several variables showed statistically significant deviations from normality based on the Shapiro–Wilk test. Therefore, Spearman rank correlations were additionally performed as sensitivity analyses to assess the robustness of the Pearson correlation findings. The inverse associations of Cognitive Restraint with energy intake (*ρ* = −0.265, *p* = 0.024), fat intake (*ρ* = −0.293, *p* = 0.012), carbohydrate intake (*ρ* = −0.288, *p* = 0.014), and sugar intake (*ρ* = −0.296, *p* = 0.012) remained statistically significant and were comparable in magnitude to the corresponding Pearson correlations. In contrast, the positive Pearson correlation between Uncontrolled Eating and fat intake (*r* = 0.247, *p* = 0.036) was not confirmed by the Spearman analysis (*ρ* = 0.210, *p* = 0.077).

The Pearson associations of SAS-2 total score with carbohydrate intake (*r* = 0.233, *p* = 0.049) and sugar intake (*r* = 0.265, *p* = 0.024) were also attenuated and were not statistically significant in the Spearman analyses (*ρ* = 0.170, *p* = 0.154 and *ρ* = 0.156, *p* = 0.191, respectively). Similarly, the positive Pearson association between Concentration Disruption and sugar intake (*r* = 0.277, *p* = 0.018) was not confirmed by the Spearman analysis (*ρ* = 0.150, *p* = 0.208).

The inverse Pearson association between resilience and sugar intake (*r* = −0.318, *p* = 0.007) was attenuated and did not remain statistically significant in the Spearman analysis (*ρ* = −0.212, *p* = 0.073). In contrast, the inverse associations between resilience and sport anxiety remained statistically significant in the Spearman analyses, including SAS-2 total score (*ρ* = −0.485, *p* < 0.001), Somatic Anxiety (*ρ* = −0.344, *p* = 0.003), Worry (*ρ* = −0.453, *p* < 0.001), and Concentration Disruption (*ρ* = −0.446, *p* < 0.001).

The positive association between BMI and Cognitive Restraint was also confirmed by the Spearman analysis (*ρ* = 0.419, *p* < 0.001).

### 3.4. Multiple Regression Analyses

Multiple linear regression analyses were conducted to determine whether eating behaviour dimensions predicted energy, macronutrient and sugar intake, both in absolute terms and, for macronutrients, as percentages of total energy intake; the results are presented in Table 4 and Table 5.

The overall regression models were significant for daily energy intake, fat intake, and carbohydrate intake. Within the significant models, higher Uncontrolled Eating significantly predicted greater daily energy intake and fat intake. Although the overall carbohydrate model was significant, none of the individual predictors reached statistical significance. The overall models for protein and sugar intake did not reach statistical significance.

Cognitive Restraint showed trend-level inverse associations with energy intake (*β* = −0.210, *p* = 0.075), fat intake (*β* = −0.227, *p* = 0.052), carbohydrate intake (*β* = −0.208, *p* = 0.081), and sugar intake (*β* = −0.213, *p* = 0.076). Emotional Eating was not significantly associated with any of the absolute dietary intake variables (all *p* > 0.05).

When macronutrient intakes were expressed as percentages of total energy intake, none of the overall regression models including Uncontrolled Eating, Cognitive Restraint, and Emotional Eating as predictors reached statistical significance for protein, fat, carbohydrate, or sugar intake (Table 5).

In additional sensitivity analyses adjusted for BMI, the overall models remained significant for daily energy intake (*R*^2^ = 0.143, *F*(4,67) = 2.80, *p* = 0.033) and absolute fat intake (*R*^2^ = 0.162, *F*(4,67) = 3.23, *p* = 0.017). Uncontrolled Eating remained a significant positive predictor of both energy intake (*β* = 0.328, *p* = 0.036) and fat intake (*β* = 0.359, *p* = 0.021). The overall models for absolute protein, carbohydrate, and sugar intake, as well as all models for macronutrient intake expressed as a percentage of total energy intake, were not statistically significant (all *p* > 0.05). BMI was not a significant predictor in any of the examined models (all *p* > 0.05).

Sport anxiety was not a significant predictor of energy intake when the SAS-2 subscales were entered into the model, although the SAS-2 total score showed a trend-level positive association (*β* = 0.222, *p* = 0.061). Additional regression analyses indicated that none of the SAS-2 subscales (Somatic Anxiety, Worry, and Concentration Disruption) significantly predicted protein, fat, carbohydrate, or sugar intake (all *p* > 0.05). Although Concentration Disruption was weakly positively correlated with sugar intake in the correlation analyses, this association did not remain significant in the regression model.

When the SAS-2 total score was examined as a single predictor, positive associations were observed with several dietary intake variables. The SAS-2 total score significantly predicted carbohydrate intake (*B* = 4.76, 95% *CI* [0.02, 9.51], *p* = 0.049), explaining 5.4% of the variance (*R*^2^ = 0.054). Trend-level positive associations were also observed for protein intake (*B* = 1.25, 95% *CI* [−0.14, 2.64], *p* = 0.077, *R*^2^ = 0.044) and fat intake (*B* = 1.70, 95% *CI* [−0.26, 3.67], *p* = 0.089, *R*^2^ = 0.041). These findings suggest that higher overall sport anxiety may be associated with greater macronutrient intake, although the observed effect sizes were small.

Resilience significantly predicted absolute sugar intake, explaining 10.1% of its variance, whereas no significant associations were observed for total energy, protein, carbohydrate, or fat intake. To examine whether this association persisted when sugar intake was considered relative to total energy intake, an additional regression analysis was performed with sugar intake expressed as a percentage of total energy intake. The negative association remained significant, with the model explaining 11.5% of the variance in sugar intake expressed as a percentage of total energy intake. The results for absolute sugar intake and sugar intake expressed as a percentage of total energy intake are presented in Table 6.

In sensitivity analyses additionally adjusting for chronological age, age was not a significant predictor in any of the examined models (all *p* > 0.05). The significant positive associations of Uncontrolled Eating with total energy intake (*β* = 0.335, *p* = 0.033) and absolute fat intake (*β* = 0.368, *p* = 0.018) remained after adjustment for age. The overall model for absolute carbohydrate intake, however, no longer reached statistical significance (*p* = 0.077). The negative association between resilience and sugar intake also remained significant after adjustment for age, both for absolute sugar intake (*β* = −0.324, *p* = 0.006) and sugar intake expressed as a percentage of total energy intake (*β* = −0.344, *p* = 0.004).

To examine whether differences between the two assessment sites/groups influenced the regression findings, the analyses were repeated with assessment site/group included as an additional covariate. Assessment site/group was not a significant predictor of energy intake (*p* = 0.196), protein intake (*p* = 0.197), fat intake (*p* = 0.167), carbohydrate intake (*p* = 0.252), or sugar intake (*p* = 0.090). The main pattern of associations was generally preserved after adjustment. Uncontrolled Eating remained a significant positive predictor of energy intake (*β* = 0.357, *p* = 0.022) and fat intake (*β* = 0.387, *p* = 0.012). In the adjusted models, Uncontrolled Eating also reached statistical significance for carbohydrate intake (*β* = 0.309, *p* = 0.049), while Emotional Eating was inversely associated with fat intake (*β* = −0.306, *p* = 0.047). The adjusted carbohydrate model was at the conventional significance threshold (*R*^2^ = 0.130, *F*(4,67) = 2.51, *p* = 0.050). For sugar intake, the overall adjusted model was significant (*R*^2^ = 0.139, *F*(4,67) = 2.70, *p* = 0.038), although none of the individual eating-behaviour dimensions reached statistical significance.

The assessment site/group was likewise not significantly associated with protein, fat, carbohydrate, or sugar intake expressed as percentages of total energy intake (all *p* > 0.05). None of these models based on intake expressed as a percentage of total energy intake reached overall statistical significance after inclusion of the assessment site/group.

## 4. Discussion

The present study aimed to examine the associations between eating behaviour characteristics, dietary intake, BMI, sport anxiety, and resilience among adolescent female handball players. To our knowledge, limited research has investigated the relationships between eating behaviours and dietary intake in adolescent athletes, particularly in female team-sport athletes. The main findings of the present study were that Uncontrolled Eating significantly predicted total energy and fat intake, whereas BMI was not a significant predictor of energy or macronutrient intake in the sensitivity analyses. In contrast, Cognitive Restraint was positively associated with BMI and inversely associated with several dietary intake variables. Higher resilience was independently associated with both lower absolute sugar intake and a lower proportion of total energy derived from sugar in the regression analyses, while resilience was not associated with eating behaviour characteristics. Resilience was also inversely associated with sport anxiety. Importantly, the main pattern of the findings was generally preserved in the sensitivity analyses adjusting for chronological age and assessment site/group, suggesting that the principal associations were not substantially explained by the relatively broad age range of the sample or by differences between the two assessment settings. Some secondary associations changed in statistical significance after adjustment, and these findings should therefore be interpreted cautiously. Overall, the results identify potentially relevant behavioural and psychological correlates of dietary intake in adolescent female handball players, while their cross-sectional and partly exploratory nature warrants cautious interpretation.

Interestingly, Uncontrolled Eating significantly predicted total energy and fat intake, whereas no significant individual effect of Uncontrolled Eating was observed for carbohydrate intake, and the overall models for protein and sugar intake did not reach statistical significance. This pattern may reflect differences in the factors associated with overall energy consumption and the intake of specific nutrients. The relatively high contribution of fat to total energy intake in the present sample (41.1 ± 3.98%) may also be relevant to this pattern. Although a relatively high proportion of energy derived from fat has previously been reported in the general Hungarian adolescent population, with fat intake exceeding the recommended level across age groups and both sexes [40], the proportion observed in the present sample was even higher. It also exceeded the generally recommended range of 25–35% of total energy intake for young athletes [41]. Given that fat provides approximately 9 kcal/g, variation in fat intake may contribute substantially to variation in total energy intake. Thus, the parallel associations of Uncontrolled Eating with total energy and fat intake may partly reflect the dietary composition observed in this sample, although this interpretation remains speculative.

From a behavioural perspective, Uncontrolled Eating reflects difficulty in limiting food intake and greater sensitivity to hunger and external food-related cues. Previous research using the TFEQ-R18 has linked higher Uncontrolled Eating to greater energy intake and to dietary patterns characterised by greater consumption of energy-dense and fatty foods, although these associations were more evident in adults than in teenagers and young adults [42]. Such behavioural susceptibility may provide a potential explanation for the positive association between Uncontrolled Eating and fat intake observed in the present sample. Thus, the observed association may reflect an interplay between Uncontrolled Eating tendencies and the dietary pattern of this specific athletic sample rather than a universal nutrient-specific effect of Uncontrolled Eating. However, given the age-related differences reported previously and the cross-sectional nature of the present study, this interpretation should be considered tentative. Although psychophysiological processes may also be relevant to associations between eating behaviour and dietary intake, these mechanisms were not directly assessed in the present study and therefore cannot be evaluated from the current data.

Previous research has indicated that sweet taste preference in a mixed-sex sample of young adults may be more strongly associated with added sugar intake than eating behaviour traits measured by the TFEQ, highlighting the potential role of sensory preferences in shaping sugar consumption [43]. In the present study, resilience was negatively associated with sugar intake, despite being unrelated to Cognitive Restraint, Uncontrolled Eating, and Emotional Eating. This finding partially aligns with results from the NutriNet-Santé study, where higher resilience was associated with healthier dietary patterns, including lower consumption of sugary foods [44]. However, unlike the NutriNet-Santé cohort, resilience was not associated with eating behaviour characteristics in the present sample, suggesting that its relationship with dietary intake may operate through different mechanisms in adolescent female athletes.

The finding that resilience predicted sugar intake despite being unrelated to eating behaviour characteristics suggests that psychological factors influencing dietary choices may extend beyond the eating behaviour dimensions captured by the TFEQ.

Previous studies have highlighted the potential role of emotional eating in shaping dietary intake. Comparable findings were reported by Anschutz et al. [13] in young adult women, who examined the associations between eating behaviour dimensions and dietary intake using structural equation modelling while controlling for BMI and physical activity. In that study, external eating was positively associated with total energy, fat, and carbohydrate intake, whereas restrained eating showed inverse associations with these dietary variables. Although a different instrument (DEBQ) was used, this pattern is partly comparable to the present findings. Cognitive Restraint showed consistent inverse bivariate associations with energy, fat, carbohydrate, and sugar intake in the present study. Importantly, these associations remained statistically significant in the Spearman sensitivity analyses, supporting the robustness of their direction despite deviations from normality in some variables. However, Cognitive Restraint did not emerge as a significant independent predictor in the regression models. Thus, the findings suggest an inverse association between Cognitive Restraint and habitual dietary intake at the bivariate level, but do not provide evidence for an independent contribution after the eating-behaviour dimensions were considered simultaneously.

Emotional Eating, however, did not show significant associations with dietary intake in either study. In the NutriNet-Santé cohort, emotional eating partly mediated the associations between psychological resilience and energy intake as well as several dietary outcomes in predominantly middle-aged adults [44]. Similarly, Lopez-Cepero et al. [45] reported positive associations between emotional eating, overeating episodes, and the consumption of energy-dense foods in a community-based sample of Latino adults. Consistent with these findings, a recent systematic review demonstrated that emotional eating is frequently associated with greater consumption of hyperpalatable energy-dense foods and less favourable indicators of nutritional status [46]. However, Emotional Eating reflects a tendency to eat in response to emotional states and may therefore be more dependent on the presence and intensity of emotional triggers. Previous evidence indicates that stress and negative affect may influence appetite and food intake differently across individuals [47]. Although dietary intake and psychological characteristics were assessed during the same assessment session in the present study, the SQFFQ captured habitual dietary intake rather than food consumption in relation to specific emotional states or episodes. Consequently, context-dependent associations between emotional states and eating may not have been fully captured. In contrast, Emotional Eating was not a significant predictor of dietary intake in the present study, whereas Uncontrolled Eating emerged as a significant predictor of total energy and fat intake. These discrepancies may reflect differences in participant characteristics, including age, sex distribution, and athletic status, suggesting that the eating behaviour dimensions most strongly related to dietary intake may vary across populations. Given the cross-sectional design and the absence of assessment of eating in relation to specific emotional episodes, these explanations remain tentative. The sensitivity power analysis should also be considered when interpreting the absence of significant associations for some eating-behaviour dimensions. With the present sample size, the three-predictor regression models had 80% power to detect effects of approximately *f*^2^ = 0.160 (corresponding to *R*^2^ ≈ 0.138), indicating that smaller effects may not have been reliably detected. Therefore, the non-significant regression coefficients, particularly for Cognitive Restraint and Emotional Eating, should not be interpreted as evidence that these dimensions are unrelated to dietary intake.

Sport anxiety was not a significant predictor of energy intake when the SAS-2 subscales were entered into the regression model. The SAS-2 total score showed a positive but non-significant association with energy intake (*β* = 0.222, *p* = 0.061). At the bivariate level, the SAS-2 total score showed weak positive Pearson correlations with carbohydrate and sugar intake and trend-level positive associations with protein and fat intake. A weak positive Pearson correlation was also observed between sugar intake and Concentration Disruption. However, the associations of SAS-2 total score with carbohydrate and sugar intake, as well as that between Concentration Disruption and sugar intake, were attenuated and no longer statistically significant in the Spearman sensitivity analyses. Thus, the observed associations between sport anxiety and dietary intake were small and not consistently supported across analytical approaches and should therefore be interpreted cautiously. Previous research has nevertheless reported associations between less favourable dietary patterns and increased anxiety symptoms among adolescents. Dietary patterns characterized by higher consumption of snack foods have been linked to a greater likelihood of anxiety symptoms in adolescent populations [48], while a recent systematic review and meta-analysis demonstrated consistent associations between higher consumption of ultra-processed foods and poorer mental health outcomes, including anxiety [49].

Resilience showed an inverse association with sugar intake in the primary analyses and was also negatively correlated with sport anxiety and all of its subdimensions. The inverse association with sugar intake was observed for both absolute sugar intake and sugar intake expressed as a percentage of total energy intake, indicating that the association was also evident when sugar intake was considered relative to total energy intake. However, the bivariate association between resilience and absolute sugar intake was attenuated and no longer statistically significant in the Spearman sensitivity analysis (*ρ* = −0.212, *p* = 0.073). Thus, although the inverse association was supported by the regression analyses, including models additionally adjusted for age, it was not fully consistent across analytical approaches and should be interpreted cautiously. In contrast, the inverse associations between resilience and sport anxiety were consistently supported by both Pearson and Spearman analyses, including SAS-2 total score and the Somatic Anxiety, Worry, and Concentration Disruption subscales.

The mean CD-RISC-10 score observed in the present sample (31.0 ± 4.8) was closely comparable to that recently reported among Hungarian youth ice hockey players of a similar age (mean age 16.19 ± 1.71 years), who had a mean item score of 3.19 ± 0.52 on the 0–4 CD-RISC-10 scale, corresponding to 31.9 ± 5.2 when expressed as a 0–40 total score [36]. In contrast, the mean score in the present sample was descriptively higher than the 26.05 ± 6.96 reported in a large Hungarian student sample from primary, secondary, and higher education in Fejér County [35]. However, these comparisons should be interpreted cautiously, as the youth ice hockey sample consisted exclusively of male athletes, while the broader Hungarian reference sample was not matched to the present sample by age, sex, or athletic status.

Although no causal relationships can be inferred, the present findings suggest that some psychological characteristics may be associated with dietary intake in adolescent athletes. In particular, resilience showed inverse associations with sugar intake across several analyses, although this finding was not fully consistent across analytical approaches. In contrast, the associations between sport anxiety and dietary intake were generally weak and were not confirmed in the Spearman sensitivity analyses. As the present study assessed sport-specific anxiety rather than general anxiety symptoms, comparisons with previous studies examining general anxiety should also be interpreted cautiously.

BMI was positively associated with Cognitive Restraint in the present study. Similar associations have previously been reported among young adults [11] and young Finnish females [50]. Moreover, Banna et al. [51] observed an almost identical positive correlation between Cognitive Restraint and BMI (*r* = 0.36, *p* < 0.001) in a sample of early adolescent girls. Consistent with these findings, a large cross-national study reported that emerging adult individuals with higher BMI tended to exhibit greater restrained eating, suggesting increased efforts to regulate food intake and body weight [52]. More recently, data from the HELENA study demonstrated that higher levels of Cognitive Restraint were associated with less favourable body composition indicators among European adolescents [17]. Taken together, these findings suggest that higher BMI, as observed in the present study, and less favourable body composition characteristics, as reported previously, may be associated with higher levels of Cognitive Restraint across different age groups. However, BMI should be interpreted with caution in athletic populations, as higher values may partly reflect greater skeletal muscle mass rather than greater adiposity. Accordingly, the positive association between BMI and Cognitive Restraint observed in the present study cannot be attributed specifically to greater adiposity. Future studies incorporating more comprehensive body composition measures may help clarify whether this association is primarily related to adiposity, muscularity, or other aspects of body composition.

In addition to its positive association with BMI, Cognitive Restraint showed inverse bivariate associations with energy, fat, carbohydrate, and sugar intake. These associations remained statistically significant in the Spearman sensitivity analyses, with comparable direction and magnitude. Similar findings have been reported among female adolescent endurance runners, where higher Cognitive Dietary Restraint scores measured using the Three-Factor Eating Questionnaire (TFEQ) significantly predicted lower energy, carbohydrate, and fat intake [53]. Furthermore, studies conducted in adults participating in weight-management interventions have demonstrated that higher dietary restraint is associated with greater dietary control and lower energy intake [54]. In the present study, however, Cognitive Restraint did not emerge as a significant independent predictor of dietary intake in the regression models. Thus, the findings indicate a consistent inverse bivariate relationship between Cognitive Restraint and habitual dietary intake but do not establish an independent association between Cognitive Restraint and intake when the eating-behaviour dimensions are considered simultaneously. Although Cognitive Restraint was associated with both BMI and dietary intake, BMI itself was not significantly associated with daily energy intake, highlighting the distinct information provided by behavioural and anthropometric measures.

In the present study, BMI did not significantly predict energy or macronutrient intake. In contrast, Uncontrolled Eating remained a significant positive predictor of total energy and fat intake after adjustment for BMI. These findings indicate that the observed associations between Uncontrolled Eating and dietary intake were not explained by BMI within the present sample. However, given the cross-sectional design, the relative contribution or direction of the relationships between eating behaviour, body size, and dietary intake cannot be determined. Previous research in adolescents has similarly demonstrated complex associations between eating behaviours, dietary outcomes, and BMI, with dietary restraint and disinhibition showing distinct relationships with diet quality and weight-related outcomes [55].

The examination of eating-related behaviours may be especially important during mid-to-late adolescence. In a sample of elite youth athletes, the highest prevalence of clinically relevant eating-disorder symptoms was observed among female athletes aged 15–18 years [56]. Given that the present sample falls within a similar age range, greater attention to eating-related behaviours may be warranted in this population. However, the eating-behaviour dimensions assessed by the TFEQ-R21 should not be interpreted as indicators of eating-disorder symptoms or clinical eating pathology.

### Strengths and Limitations

Several limitations of the present study should be acknowledged. First, the cross-sectional design precludes any conclusions regarding causality or the direction of the observed associations. Second, dietary intake was assessed using a semi-quantitative food frequency questionnaire (SQFFQ), which is based on self-report and therefore may be subject to recall bias and estimation errors. Although the SQFFQ was specifically developed to reflect dietary patterns and food products relevant to the Hungarian population, it was not specifically developed or validated for use in athletic populations, and the specific 90-item version used in the present study has not been formally validated against an independent reference dietary assessment method. This should be considered when interpreting the estimated energy and nutrient intakes.

Furthermore, although brief or short-form psychological instruments were selected where appropriate and participants completed the assessments without time pressure, the overall questionnaire burden, including the 90-item SQFFQ, may have contributed to response fatigue and potentially affected response accuracy. Although established psychological instruments were used, the Hungarian version of the SAS-2 has not been formally validated, which should be considered when interpreting the sport anxiety findings.

In addition, information on menstrual status and menstrual cycle characteristics was not collected, although hormonal fluctuations may influence both psychological characteristics and dietary behaviours in adolescent female athletes. Furthermore, body composition data were available only for a subsample of participants, limiting the examination of associations involving body composition variables. The use of convenience sampling, the relatively small sample size, the inclusion of athletes from a single sport, and the absence of comparison groups comprising non-athletes, athletes from other sports, or male athletes may limit the generalizability of the findings. Consequently, it cannot be determined whether the observed associations are specific to adolescent female handball players or are also present in other athletic or non-athletic populations. Future studies with larger samples could investigate whether distinct eating behaviour profiles can be identified based on combinations of Uncontrolled Eating, Emotional Eating, and Cognitive Restraint. Such profile-based approaches may help determine whether subgroups differ in BMI, body fat, skeletal muscle mass, sport anxiety, resilience, and dietary intake.

From a methodological perspective, the relatively small sample size and the number of correlation and regression analyses performed should be considered when interpreting the findings. Sensitivity power analyses indicated that the study was primarily able to detect moderate or larger associations, and smaller effects may therefore have remained undetected. Consequently, non-significant findings should not necessarily be interpreted as evidence of the absence of an association. Conversely, no formal adjustment for multiple testing was applied; therefore, the possibility of type I error cannot be excluded, particularly for associations with *p*-values close to the conventional significance threshold. Accordingly, these findings should be regarded as exploratory until replicated in larger independent samples. Several variables also deviated from normality, and although Spearman sensitivity analyses confirmed some of the principal correlation patterns, several weaker associations identified using Pearson correlations were attenuated and no longer statistically significant. Although regression assumptions were generally met, the residuals of some models deviated from normality according to the Shapiro–Wilk test. However, given the sample size and the absence of substantial multicollinearity or influential outliers, the regression analyses were considered sufficiently robust. These methodological considerations warrant cautious interpretation of the findings.

Despite these limitations, the present study has several strengths. To our knowledge, this is one of the first studies to simultaneously examine eating behaviour characteristics, sport anxiety, resilience, and dietary intake in adolescent elite female handball players.

The study focused on a specific and underrepresented athletic population and employed established psychological instruments, providing novel insights into the behavioural and psychological correlates of dietary intake in adolescent female athletes.

## 5. Conclusions

In conclusion, the present findings indicate that eating behaviour dimensions are associated with dietary intake in adolescent elite female handball players, although the pattern of associations differed across eating behaviour dimensions. Uncontrolled Eating was a significant positive predictor of total energy and fat intake, whereas Cognitive Restraint showed consistent inverse bivariate associations with energy, fat, carbohydrate, and sugar intake and was positively associated with BMI. Resilience showed an inverse association with sugar intake across several analyses, although this finding was not consistent across all sensitivity analyses, while its inverse associations with sport anxiety were more consistent. Overall, these findings highlight the potential relevance of considering behavioural and psychological characteristics alongside dietary assessment in adolescent female athletes. Given the cross-sectional design and the exploratory nature of the analyses, these findings warrant cautious interpretation and replication in larger independent samples.

## Figures and Tables

**Table 1:** Participant Characteristics (*N* = 72).

Variable	Mean ± *SD*
Age (years)	15.6 ± 1.7
Height (cm)	168.0 ± 6.5
Body weight (kg)	60.2 ± 8.8
BMI (kg/m^2^)	21.2 ± 2.4
Body fat (%) ^a^	19.9 ± 5.8
Skeletal muscle mass (kg) ^a^	25.3 ± 3.1
Handball training (h/week)	8.76 ± 1.4
Other sports training (h/week)	0.13 ± 0.4
Physical education classes (h/week)	2.38 ± 1.0

Note. Values are presented as mean ± *SD*. ^a^ Available for 38 participants. All other variables were available for 72 participants.

**Table 2:** Dietary intake and psychological characteristics.

Variable	Mean ± *SD*
Dietary intake	
Energy (kcal/day)	3401.0 ± 1448.0
Fat (g/day)	151.0 ± 66.8
Carbohydrate (g/day)	372.0 ± 162.0
Sugar (g/day)	88.3 ± 57.6
Protein (g/day)	114.0 ± 47.2
Eating behaviour (TFEQ-R21) (points)	
Uncontrolled Eating (UE)	34.1 ± 18.2
Cognitive Restraint (CR)	39.2 ± 23.3
Emotional Eating (EE)	21.7 ± 19.4
Psychological characteristics (points)	
SAS-2 total score	31.6 ± 7.9
Somatic Anxiety	8.63 ± 2.9
Worry	14.5 ± 4.2
Concentration Disruption	8.46 ± 2.3
CD-RISC-10 total score	31.0 ± 4.8

Note. TFEQ-R21, Three-Factor Eating Questionnaire-Revised 21-item; SAS-2, Sport Anxiety Scale-2; CD-RISC-10, Connor-Davidson Resilience Scale-10.

**Table 3:** Correlations between dietary intake, eating behaviour, sport anxiety and resilience variables. * *p* < 0.05, ** *p* < 0.01, *** *p* < 0.001.

Variable	1	2	3	4	5	6	7	8	9	10	11	12
1. Energy intake (kcal/day)	—											
2. Fat intake (g/day)	0.982 ***	—										
3. Carbohydrate intake (g/day)	0.981 ***	0.928 ***	—									
4. Sugar intake (g/day)	0.807 ***	0.771 ***	0.840 ***	—								
5. Protein intake (g/day)	0.960 ***	0.942 ***	0.919 ***	0.680 ***	—							
6. Uncontrolled Eating	0.223	0.247 *	0.200	0.172	0.185	—						
7. Cognitive Restraint	−0.266 *	−0.288 *	−0.257 *	−0.255 *	−0.183	−0.202	—					
8. Emotional Eating	−0.013	−0.005	−0.007	−0.016	−0.057	0.649 ***	−0.048	—				
9. Somatic Anxiety	0.189	0.166	0.200	0.224	0.192	0.174	0.153	0.094	—			
10. Worry	0.194	0.189	0.192	0.195	0.190	0.188	−0.048	0.120	0.540 ***	—		
11. Concentration Disruption	0.174	0.145	0.203	0.277 *	0.139	0.164	−0.013	0.096	0.672 ***	0.514 ***	—	
12. Resilience	−0.151	−0.120	−0.195	−0.318 **	−0.075	−0.169	−0.027	−0.127	−0.475 ***	−0.434 ***	−0.541 ***	—

**Table 4:** Multiple linear regression analyses predicting absolute dietary intake from eating behaviour dimensions.

Outcome	Predictor	*β*	*p*	Model *R*^2^	*F*(3,68)	Model *p*
Energy intake (kcal/day)	UE	**0.338**	**0.030**	**0.134**	**3.51**	**0.020**
	CR	−0.210	0.075			
	EE	−0.242	0.110			
Protein intake (g/day)	UE	0.347	0.028	0.104	2.62	0.058
	CR	−0.127	0.287			
	EE	−0.288	0.062			
Fat intake (g/day)	UE	**0.366**	**0.017**	**0.157**	**4.23**	**0.008**
	CR	−0.227	0.052			
	EE	−0.254	0.090			
Carbohydrate intake (g/day)	UE	0.292	0.063	**0.113**	**2.89**	**0.042**
	CR	−0.208	0.081			
	EE	−0.206	0.178			
Sugar intake (g/day)	UE	0.252	0.108	0.101	2.54	0.064
	CR	−0.213	0.076			
	EE	−0.190	0.217			

Note. UE = Uncontrolled Eating; CR = Cognitive Restraint; EE = Emotional Eating. *β* = standardised regression coefficient. Significant associations (*p* < 0.05) are shown in bold.

**Table 5:** Multiple linear regression analyses predicting macronutrient and sugar intake expressed as percentages of total energy intake from eating behaviour dimensions.

Outcome	Predictor	*β*	*p*	Model *R*^2^	*F*(3,68)	Model *p*
Protein (% energy)	UE	0.025	0.871	0.105	2.67	0.055
	CR	0.305	0.012			
	EE	−0.122	0.423			
Fat (% energy)	UE	0.149	0.356	0.041	0.98	0.409
	CR	−0.141	0.252			
	EE	−0.142	0.370			
Carbohydrate (% energy)	UE	−0.139	0.395	0.018	0.41	0.745
	CR	0.014	0.909			
	EE	0.167	0.297			
Sugar (% energy)	UE	0.033	0.838	0.044	1.03	0.385
	CR	−0.196	0.113			
	EE	−0.080	0.612			

Note. UE = Uncontrolled Eating; CR = Cognitive Restraint; EE = Emotional Eating. *β* = standardised regression coefficient. None of the overall regression models were statistically significant at *p* < 0.05.

**Table 6:** Linear regression analyses of resilience predicting absolute sugar intake and sugar intake expressed as a percentage of total energy intake.

	Sugar Intake (g/day)	Sugar Intake (% Energy)
Predictor	CD-RISC-10	CD-RISC-10
*β*	**−0.318**	**−0.340**
*p*	**0.007**	**0.004**
*R* ^2^	**0.101**	**0.115**
*F*(1,70)	**7.85**	**9.14**
Model *p*	**0.007**	**0.004**

Note. *β* = standardised regression coefficient; CD-RISC-10 = 10-item Connor–Davidson Resilience Scale. Sugar intake was analysed both as absolute intake (g/day) and as a percentage of total energy intake. Significant associations (*p* < 0.05) are shown in bold.

## Data Availability

The datasets generated and/or analysed during the current study are not publicly available because they contain sensitive data collected from human participants, including minors. Although the data were coded and did not contain direct personal identifiers, public data sharing was not covered by the informed consent provided by the participants and their parents or legal guardians. De-identified data may be made available from the corresponding author on reasonable request, in accordance with applicable data protection regulations and institutional policies.

## Abbreviations

The following abbreviations are used in this manuscript:

BMI	Body Mass Index
CD-RISC-10	10-item Connor-Davidson Resilience Scale
CI	Confidence Interval
CR	Cognitive Restraint
DEBQ	Dutch Eating Behaviour Questionnaire
EE	Emotional Eating
HELENA	Healthy Lifestyle in Europe by Nutrition in Adolescence
IQR	Interquartile Range
kcal	Kilocalorie
R^2^	Coefficient of Determination
SAS-2	Sport Anxiety Scale-2
SD	Standard Deviation
SQFFQ	Semi-Quantitative Food Frequency Questionnaire
TFEQ-R21	Three-Factor Eating Questionnaire-Revised 21-item
UE	Uncontrolled Eating
VIF	Variance Inflation Factor
